# Simultaneous ^13^N-Ammonia and gadolinium first-pass myocardial perfusion with quantitative hybrid PET-MR imaging: a phantom and clinical feasibility study

**DOI:** 10.1186/s41824-019-0062-6

**Published:** 2019-09-03

**Authors:** Muhummad Sohaib Nazir, Sarah-May Gould, Xenios Milidonis, Eliana Reyes, Tevfik F. Ismail, Radhouene Neji, Sébastien Roujol, Jim O’Doherty, Hui Xue, Sally F. Barrington, Tobias Schaeffter, Reza Razavi, Paul Marsden, Peter Kellman, Sven Plein, Amedeo Chiribiri

**Affiliations:** 1 Department of Cardiovascular Imaging, School of Biomedical Engineering and Imaging Sciences, King’s College London, St Thomas’ Hospital, Westminster Bridge, 4th Floor Lambeth Wing, London, SE1 7EH UK; 2 Siemens Healthcare Limited, Sir William Siemens Square, Frimley, Camberley, GU16 8QD UK; 3 National Heart, Lung, and Blood Institute, National Institutes of Health, DHHS, Bethesda, MD USA; 4 Physikalisch-Technische Bundesanstalt, Berlin, Germany; 5 Leeds Institute of Cardiovascular and Metabolic Medicine, LIGHT Laboratories, Clarendon Way, University of Leeds, Leeds, LS2 9JT UK

**Keywords:** Hybrid imaging, PET-MR, Myocardial perfusion, Myocardial blood flow

## Abstract

**Background:**

Positron emission tomography (PET) is the non-invasive reference standard for myocardial blood flow (MBF) quantification. Hybrid PET-MR allows simultaneous PET and cardiac magnetic resonance (CMR) acquisition under identical experimental and physiological conditions. This study aimed to determine feasibility of simultaneous ^13^N-Ammonia PET and dynamic contrast-enhanced CMR MBF quantification in phantoms and healthy volunteers.

**Methods:**

Images were acquired using a 3T hybrid PET-MR scanner. Phantom study: MBF was simulated at different physiological perfusion rates and a protocol for simultaneous PET-MR perfusion imaging was developed. Volunteer study: five healthy volunteers underwent adenosine stress. ^13^N-Ammonia and gadolinium were administered simultaneously. PET list mode data was reconstructed using ordered subset expectation maximisation. CMR MBF was quantified using Fermi function-constrained deconvolution of arterial input function and myocardial signal. PET MBF was obtained using a one-tissue compartment model and image-derived input function.

**Results:**

Phantom study: PET and CMR MBF measurements demonstrated high repeatability with intraclass coefficients 0.98 and 0.99, respectively. There was high correlation between PET and CMR MBF (*r* = 0.98, *p* < 0.001) and good agreement (bias − 0.85 mL/g/min; 95% limits of agreement 0.29 to − 1.98). Volunteer study: Mean global stress MBF for CMR and PET were 2.58 ± 0.11 and 2.60 ± 0.47 mL/g/min respectively. On a per territory basis, there was moderate correlation (*r* = 0.63, *p* = 0.03) and agreement (bias − 0.34 mL/g/min; 95% limits of agreement 0.49 to − 1.18).

**Conclusion:**

Simultaneous MBF quantification using hybrid PET-MR imaging is feasible with high test repeatability and good to moderate agreement between PET and CMR. Future studies in coronary artery disease patients may allow cross-validation of techniques.

**Electronic supplementary material:**

The online version of this article (10.1186/s41824-019-0062-6) contains supplementary material, which is available to authorized users.

## Background

Positron emission tomography (PET) is considered the non-invasive reference standard for the quantification of absolute myocardial blood flow (MBF) (Bratis et al. 2013). Quantification with PET has proven diagnostic (Farhad et al. 2013) and prognostic value in patients with coronary artery disease (CAD) (Herzog et al. 2009). Quantification of MBF is also feasible with CMR (Jerosch-Herold 2010), although it often involves time-consuming and expert post-processing analysis that has hampered widespread uptake in clinical routine. Fully automated methods for CMR-derived MBF quantification with free breathing, motion corrected methods are now available and have been tested in healthy volunteers with good reproducibility (Brown et al. 2018) and agreement with ^13^N-Ammonia PET (Engblom et al. 2017). In recent observational studies, fully automated CMR pixel-wise MBF measurements showed good diagnostic accuracy in patients with suspected CAD (Hsu et al. 2018; Knott et al. 2019; Kotecha et al. 2019).

Previous studies that have compared MBF quantification between CMR and PET have focussed on separate assessments of PET and CMR performed on common groups of subjects but separated in time and thus being subject to intrasubject variation (Morton et al. 2012a; Miller et al. 2014; Fritz-Hansen et al. 2008; Parkka et al. 2006). The largest comparative study showed good agreement for myocardial perfusion reserve (ratio of stress MBF:rest MBF) between PET and CMR, but poor agreement for absolute MBF values (Morton et al. 2012b). Prior to adoption of quantitative CMR MBF in clinical practice, it is important to understand the causes for discrepant findings. The interstudy variation for stress MBF has been reported to be 19% with PET (Kitkungvan et al. 2017) and 12–40% with CMR (Brown et al. 2018; Larghat et al. 2013). Hence, it is unknown how much variation relates to intrinsic differences in modelling, imaging and tracer kinetics or due to biological variation. Intrasubject biological variation between PET and MR measurements can be avoided by performing simultaneous PET and CMR imaging that provides an opportunity to ascertain the intrinsic magnitude of differences in MBF measurements without physiological variation (Nazir et al. 2018).

The purpose of this study was to demonstrate the feasibility of simultaneous CMR and PET perfusion quantification with hybrid PET-MR imaging under identical experimental and physiological conditions ex vivo and in vivo.

## Methods

All data were acquired on a 3T hybrid PET-MR scanner (Biograph mMR, Siemens Healthcare, Erlangen, Germany) with integrated PET and MRI systems housed in the same gantry. CMR data were acquired with a flexible anterior 6-channel body transmission coil arrayed receiver, and posterior 6-channel spine arrayed receiver mounted in the scanner table.

### Phantom study

We have previously described a physical perfusion phantom that mimics the cardiac chambers, great thoracic vessels and myocardium, making it possible to simulate first-pass myocardial perfusion with high reproducibility (Chiribiri et al. 2013). In this current study, a modified, 3D-printed version of the phantom has been used, which has the potential to allow widespread validation and calibration across sites and different imaging modalities. Furthermore, preclinical evaluation of methodology and quantification algorithms is possible in a controlled environment devoid of respiratory or motion artefact. Details of the perfusion phantom are provided in Additional file 1.

Using a fixed cardiac output and heart rate of 60 beats per minute, a range of myocardial perfusion rates were generated from 1 to 5 mL/g/min in 1 mL/g/min increments. Radiotracer and gadolinium contrast were administered directly into the inferior vena cava of the perfusion phantom via a power injector (Spectris Solaris, Medrad®, Bayer AG) at a rate of 4 mL/s.

After completion of each experimental run, flow rates of the phantom were increased to the maximum level for a minimum of 5 min in order to allow complete washout of residual ^13^N-Ammonia and gadolinium contrast from the entire phantom circuitry. As such, steady and repeatable experimental conditions were created. Knowledge of the dispersion volume at site of sampling of the tissue compartment allowed MBF values to be derived in units of mL/g/min for measurements made with both modalities.

**Fig. 1:**
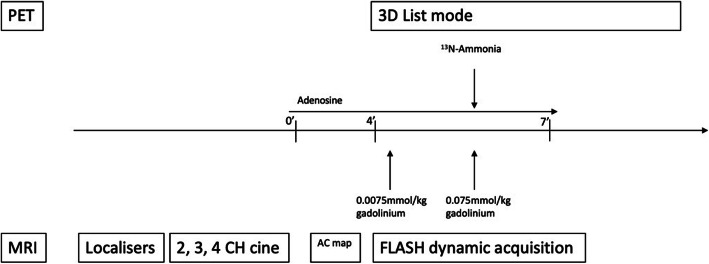
Protocol for simultaneous PET-MR perfusion imaging. ^13^N-Ammonia was administered simultaneously with the neat bolus of gadolinium contrast. Cine imaging or adenosine was not acquired/administered for the phantom study. *AC* attenuation correction, *CH* chamber, *SR*-*FLASH* saturation recovery prepared fast low angle shot

Gadobutrol (Gadovist, Bayer, Germany) contrast was administered as a dual bolus with dose 0.0075 mmol/kg (dilute) + 0.075 mmol/kg (neat) as previously described (Ishida et al. 2011; Hussain et al. 2012). The predilute bolus allows the determination of the arterial input function (AIF), avoiding signal saturation effects (Jerosch-Herold 2010). A 120-s delay between the dilute and neat bolus of contrast allowed for complete washout of the predilute gadolinium contrast from the phantom circuitry before the arrival of the neat bolus of contrast agent. Then, 200 MBq ^13^N-Ammonia was administered at the same time as the neat bolus gadolinium contrast through the same intravenous line after preloading into a long line to accommodate both PET tracer and gadolinium contrast. The whole PET-MR perfusion measurement procedure was repeated once in order to determine its repeatability. The protocol for the phantom study is illustrated in Fig. 1.

CMR perfusion data were acquired using a saturation recovery sequence Fast Low Angle Shot (FLASH) imaging readout over 240 dynamics. CMR parameters were FOV 360 × 270 mm, pixel size 2.3 × 1.9 mm, slice thickness 8 mm, TI 100 ms, TR 1.9 ms, TE 0.99 ms, flip angle 10°, GRAPPA acceleration factor 2, bandwidth 1002 Hz/px. PET data were acquired in 3D list mode for 7 min following radiotracer administration. A 3D T1-weighted Dixon sequence was acquired prior to each PET acquisition and used to generate an attenuation correction (AC) map required to generate attenuation corrected PET data. PET list mode data were binned into 60 × 3 s followed by 8 × 15 s consecutive time frames for subsequent kinetic analysis. All PET frames were reconstructed using the ordered subsets expectation maximization (OSEM) algorithm with two iterations and 21 subsets using a 4-mm smoothing filter and matrix size of 344 × 344.

### Image analysis and perfusion quantification

CMR dynamic perfusion data were analysed in Osirix (OsiriX 64-bit, version 8.0.2, Pixmeo SARL, Geneva, Switzerland). A circular region of interest (ROI) with diameter 16 mm was placed around the aortic vessel in order to derive the AIF, and another ROI was placed around the myocardium compartment of the phantom (diameter 40 mm) in the same plane at a specified slice position of the myocardium with known volume of dispersion.

CMR-derived quantitative MBF values were quantified using Fermi-constrained deconvolution of the AIF and the myocardial signal intensity as previously described (Jerosch-Herold 2010; Jerosch-Herold et al. 1998), using in house software on Matlab (MathWorks®, Natick, Massachusetts, USA).

Precise co-registration of PET and MR images was achieved by dedicated fusion software—PMOD (v3.7, PMOD Technologies, Zurich, Switzerland). ROIs were placed over the aorta for determination of the arterial input function and the myocardium.

PET-derived quantitative MBF values were calculated using a well-known simple one-tissue compartment model described by De Grado et al. (1996) that was applied to both phantom and human studies using PMOD software. In the perfusion phantom, unlike tissue, there is no trapping of ^13^N-Ammonia and also no requirement for metabolite correction.

### Volunteer study

The practical setup, administration of radiotracer and image acquisition utilised in the phantom study was used to guide the clinical protocol for the volunteer study. In particular, the phantom study was used to determine the feasibility of administration of gadolinium contrast using a dual bolus technique with simultaneous administration of PET tracer.

### Study population

Healthy volunteers (*n* = 5) were prospectively recruited to undergo a single cardiac PET-MR scan. Exclusion criteria were age of less than 50 years, pregnancy and contraindication to gadolinium contrast or MRI. In addition, volunteers were carefully screened prior to enrolment into the study to ensure absence of symptoms suggestive of cardiac disease (such as chest pain, breathlessness, syncope) and were excluded if there was a history of medical conditions, prior myocardial infarction or ischaemic heart disease, presence of any cardiovascular risk factors (hypertension, hypercholesterolaemia, diabetes mellitus, smoking history) or family history of premature myocardial infarction. All volunteers underwent a rest 12-lead ECG and were specifically asked to avoid caffeinated beverages for 24 h prior to the scan. The study was approved by the National Research Ethics Service (16/WA/0271) and the United Kingdom Administration of Radioactive Substances Advisory Committee (ARSAC) with written informed consent obtained from all patients for inclusion in the study.

### Study protocol

The cardiac PET-MR protocol is shown in Fig. 1. Following acquisition of localiser images, cine images in two chamber, four chamber and short axis (basal mid, apical) orientation are acquired using the ‘3 of 5’ rule (Plein et al. 2015). A free breathing, motion corrected saturation recovery FLASH pulse sequence was used (Kellman et al. 2017). Typical sequence parameters included FOV 360 × 270 mm, TR 1.5 ms, TE 1.0 ms, TI 115 ms, flip angle 14°, slice thickness 8 mm, pixel size 2.4 × 1.9 mm, bandwidth 1085 hz/px over 120 dynamics. Gadolinium was administered intravenously using a dual bolus approach, as in the phantom experiment, with eight baseline dynamics prior to contrast administration and 25-s interval between predilute and neat gadolinium contrast agent and was injected at 4 mL/s with a power injector following a 25 mL saline flush, also injected at 4 mL/s. 550 MBq of ^13^N-Ammonia was injected at precisely the same time as the administration of the neat gadolinium contrast agent.

For the stress section of the protocol, adenosine was administered at 140 μg/kg/min for 4 min prior to acquisition of PET and CMR data and continued for a further 3 min following radiotracer and contrast administration. Blood pressure, heart rate and symptoms were evaluated during administration of adenosine. Following acquisition of stress perfusion data, short axis cine imaging and late gadolinium enhancement (LGE) images were acquired.

PET data were acquired in 3D list mode. AC maps were acquired using a Dixon MRI sequence (Martinez-Moller et al. 2009), and following manual alignment of the AC map and PET data to ensure no respiratory misalignment of data (Lassen et al. 2017), AC-corrected PET data was reconstructed using OSEM with two iterations and 21 subsets, with a 4-mm smoothing Gaussian filter and a matrix size of 344 × 344.

### Image analysis and perfusion quantification

All PET data were quantified by an experienced PET operator with more than 10 years’ experience with cardiac PET imaging using PMOD software and blinded to the CMR quantification results. CMR data was quantified and analysed by an operator with more than 4 years’ experience in CMR and blinded to the PET quantification results. PET and CMR MBF values were obtained on a global basis, and on a territory basis of the main epicardial arteries based on 16 standard American Heart Association segmentation (Cerqueira et al. 2002).

### Statistical analysis

All statistical analyses were performed using SPSS Statistics 23 (IBM, Armonk, NY, USA). Results are expressed as mean ± standard deviation unless otherwise specified. Correlation between PET and CMR MBF were determined by Pearson’s correlation and intraclass coefficients. Agreement was determined using Bland and Altman analysis. All statistical tests were two-tailed and *p* values < 0.05 were considered significant.

## Results

### Phantom study

**Fig. 2:**
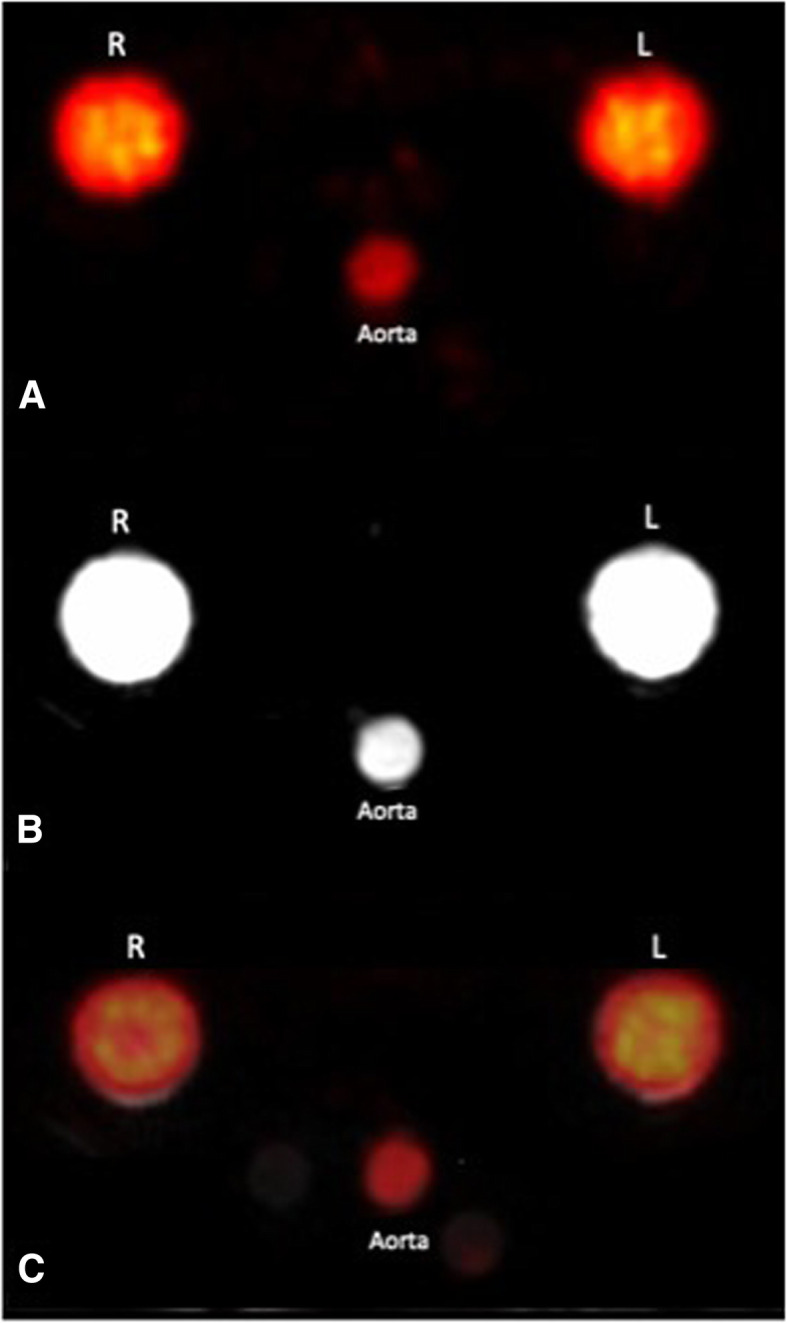
Axial images of **a** PET only, **b** CMR only and **c** hybrid PET-MR images during passage of ^13^N-Ammonia and gadolinium contrast in the perfusion phantom. *R* right myocardial compartment, *L* left myocardial compartment

All phantom experiments were completed successfully and simultaneous administration of radiotracer and gadolinium contrast and image acquisition was achieved as demonstrated in Fig. 2.

PET MBF measurements were highly repeatable with intraclass coefficients of 0.98. CMR MBF measurements were highly repeatable with intraclass coefficients of 0.99.

**Fig. 3:**
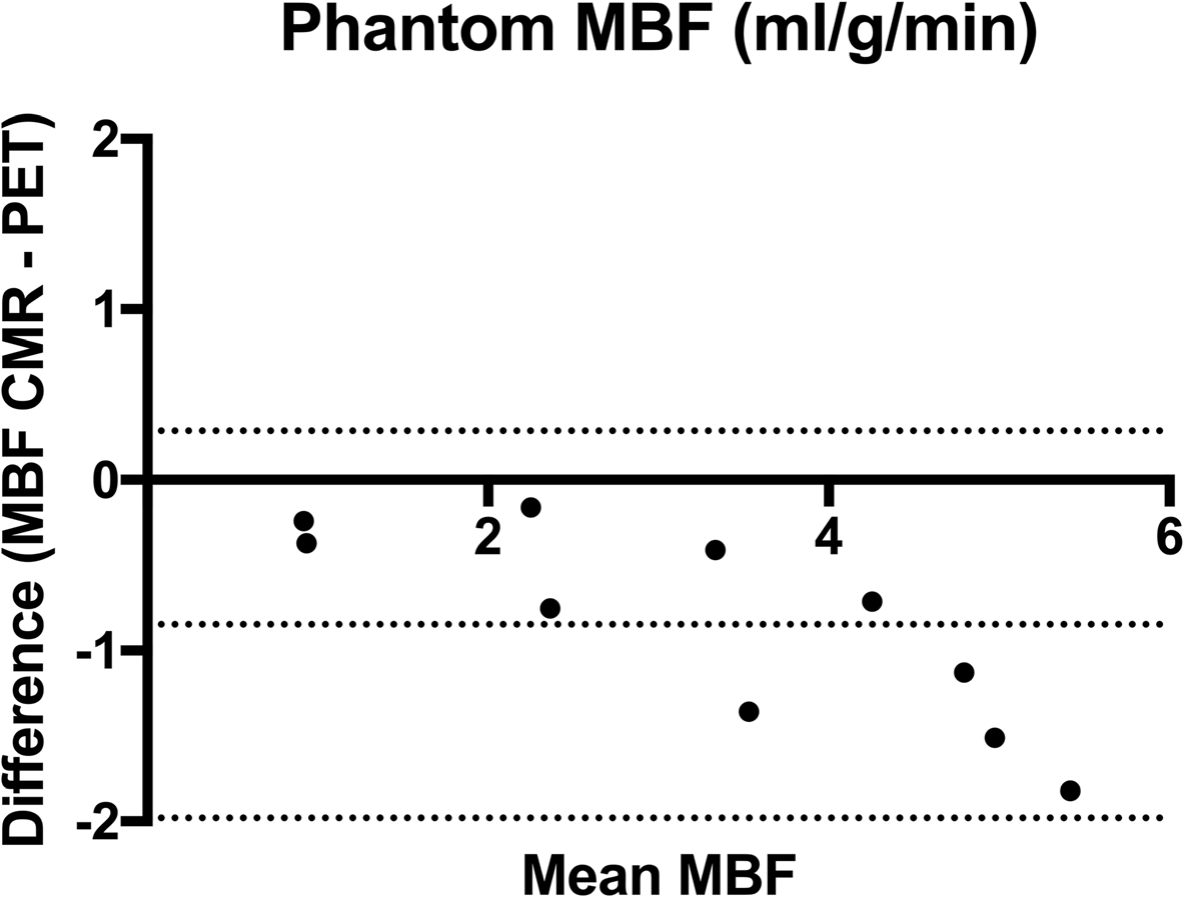
Bland and Altman plot of phantom MBF. CMR MBF underestimated PET MBF with a bias of − 0.85 mL/g/min (95% limits of agreement + 0.29 to − 1.98). Dashed lines indicate 95% limits of agreement and bias

There was a very high correlation between PET and CMR MBF measurements acquired using the perfusion phantom (*r* = 0.98, *p* < 0.001). CMR underestimated PET MBF with a bias of − 0.85 mL/g/min; 95% limits of agreement 0.29 to − 1.98 (Fig. 3). Intraclass coefficient between PET and CMR MBF was 0.91.

### Volunteer study

Participant characteristics of the volunteer cohort are shown in Table 1. One volunteer study could not be completed due to technical issues related to the scanner and therefore four patients completed the study. The CMR study demonstrated normal biventricular volumes and no evidence of scar on LGE imaging in all volunteers. The mean administered activity of ^13^N-Ammonia was 488 ± 43 MBq.

**Table 1:** Volunteer characteristics

Age	54 ± 3.5
Gender	M (100%)
BMI (kg/m^2^)	25.5 ± 3.3
Stress HR (bpm)	93 ± 15
Rest HR (bpm)	66 ± 11
Stress systolic BP (mmHg)	140 ± 2.5
Rest systolic BP (mmHg)	132 ± 12
LVEDVi (mL/m^2^)	82.5 ± 27.6
LVEF (%)	61.8 ± 4.1
LGE scar (*n*)	0

*BMI* body mass index, *HR* heart rate, *BP* blood pressure, *BPM* beats per minute, *LVEDVi* Indexed left ventricular end diastolic volume, *LVEF* left ventricular ejection fraction, *LGE* late gadolinium enhancement

**Fig. 4:**
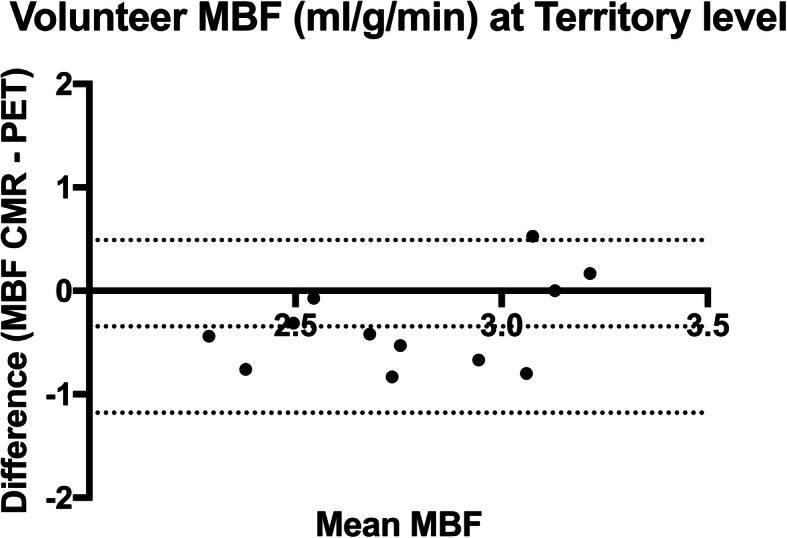
Bland and Altman plot of territorial MBF. CMR MBF underestimated PET MBF with a bias of − 0.34 mL/g/min (95% limits of agreement 0.49 to − 1.18). Dashed lines indicate 95% limits of agreement and bias

On a per patient basis, global stress MBF for CMR and PET were 2.58 ± 0.11 mL/g/min and 2.60 ± 0.47 mL/g/min respectively. On a per territory basis, there was a moderate correlation between CMR and PET MBF (*r* = 0.63, *p* = 0.03). CMR underestimated PET with a bias of − 0.34 mL/g/min (95% limits of agreement 0.49 to − 1.18) using Bland and Altman analysis as shown in Fig. 4.

**Fig. 5:**
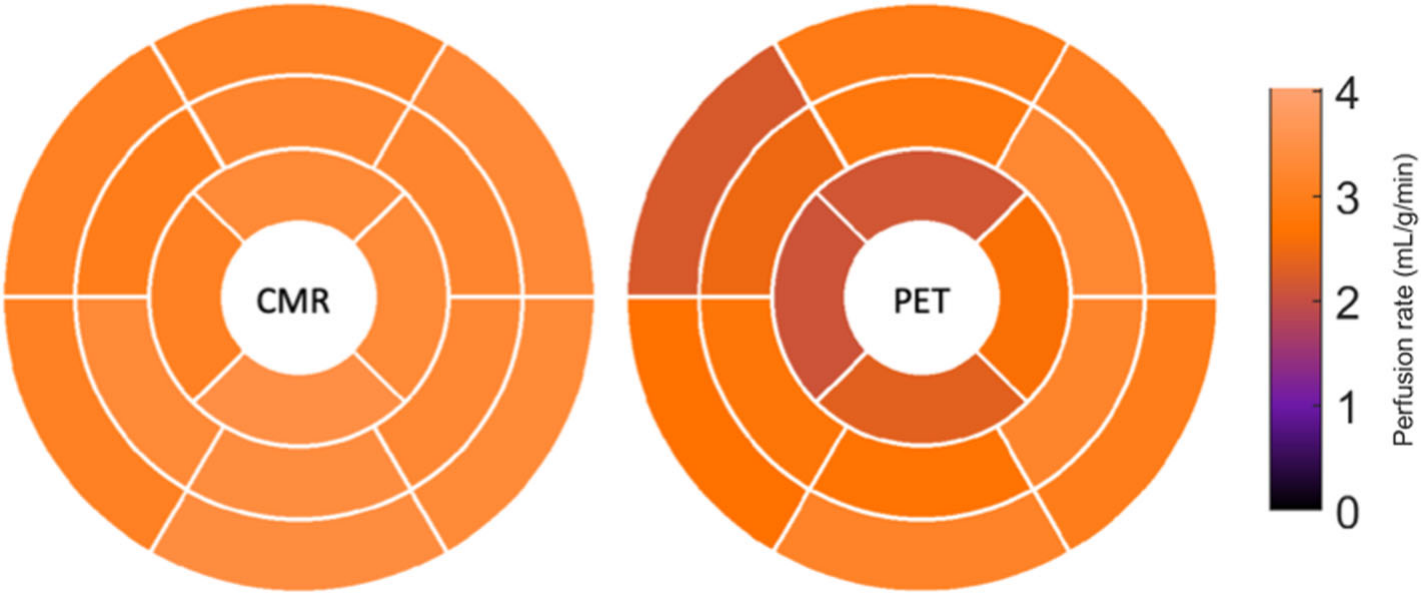
Perfusion PET-MR data from volunteer one. Side by side polar maps of MBF values with the same look up table on a 16 American Heart Association (AHA) segments

There was moderate correlation between PET and CMR territorial MBF values with intraclass correlation coefficients of 0.67. MBF perfusion maps were generated in all cases for CMR and PET. An example of PET and CMR perfusion maps for one of the volunteers are shown in Fig. 5.

## Discussion

In this study, the feasibility of simultaneous PET and CMR MBF quantification was demonstrated in a perfusion phantom and in a group of healthy volunteers. There was moderate correlation and agreement between PET and CMR MBF values. This study paves the way for a future clinical study to compare PET and CMR MBF values in a cohort of patients with suspected CAD.

The perfusion phantom served as a precursor to devise and guide a complex protocol prior to clinical hybrid imaging in a cohort of volunteers. This protocol required precise timing and delivery of perfusion radiotracers and gadolinium contrast in a safe and controlled manner, as a prerequisite to a human study. The perfusion phantom has previously been used in various studies and imaging modalities that included the investigation of different quantification algorithms for CMR-derived MBF values (Zarinabad et al. 2012), computed tomography (CT)-derived MBF values (Otton et al. 2013) and PET-MR-derived values to determine the effect of high counts rates of radiotracer (O’Doherty et al. 2017). This current study extends the utility of the perfusion phantom to demonstrate the feasibility and protocol development of fully quantitative PET and CMR-derived MBF simultaneously.

This is the first reported study in which simultaneous stress PET and CMR perfusion acquisition was undertaken in a group of healthy volunteers using hybrid PET-MR imaging. MBF values were generated within the normal ranges reported in the literature for healthy subjects, both for CMR (Brown et al. 2018) and for PET (Kitkungvan et al. 2017; Chang et al. 2018). A previous study utilized PET-MR perfusion in patients with coronary artery disease, although used a dual sequence rather than a dual bolus approach and did not have any preceding phantom studies (Kunze et al. 2018).

In this current study, CMR-derived MBF values underestimated PET values in both the phantom and volunteer studies, and these findings are in keeping with previous clinical studies that compared PET and CMR-derived MBF (Fritz-Hansen et al. 2008; Parkka et al. 2006). There are several plausible reasons to explain the underestimation of CMR-derived MBF values observed in the phantom studies and the volunteer study.

A non-linear relationship exists between MRI signal intensity and gadolinium concentration (Jerosch-Herold 2010). Mathematical derivation of MBF for CMR using Fermi deconvolution requires the arterial input and myocardial output signals to behave in the same linear relationship to that of the gadolinium concentration (Lee and Johnson 2009). In the phantom study, signal saturation effects were alleviated by indirectly normalizing the estimated impulse response functions, as native T1 values were not precisely known (Hsu et al. 2018; Zierler 1962). Specifically, the AIF was scaled so that its time-integral matched that of the mean myocardial curve. In the volunteer study, the signal intensity was converted to gadolinium concentration using a look-up table calculated by a Bloch simulation of the specific acquisition protocol used, as previously described (Kellman et al. 2017). The quantification of MBF with PET does not suffer from this non-linear relationship as the activity measured directly correlates with tracer concentration.

CMR-derived MBF can underestimate MBF without a proper correction for the non-linearity of the AIF from the blood pool (Jerosch-Herold 2010). This non-linear effect is minimized by the use of a dual bolus (Christian et al. 2004) or dual sequence (Gatehouse et al. 2004) method. In this study, we used dual bolus to correct for the non-linear AIF relationship, although undertaking such a protocol is technically challenging. A dual sequence approach (Kellman et al. 2017; Gatehouse et al. 2004), where the AIF is extracted from a low resolution FLASH readout, can be used to correct for non-linearity and may be an alternative and more practical approach that may facilitate clinical implementation.

An additional explanation for the underestimation observed with MRI is that different analysis models are used in PET and MR. Quantification of absolute MBF with CMR was achieved using a model independent approach, using a Fermi deconvolution of the AIF and myocardial signal intensity. This is based on the central volume principle (Zierler 1962), which allows calculation of flow based on the concentration of a tracer that enters and exits a particular system. However, this model does not consider the behaviour of gadolinium contrast in vivo. As such, tracer kinetic modelling methods for quantification have been described (Bassingthwaighte et al. 1989) and may provide a more accurate estimation of absolute MBF. Such an approach could not be applied to the single compartmental model phantom and thus was not used for calculation of MBF in this study, but will be the focus of future work with multicompartmental perfusion models.

PET is considered the non-invasive reference standard for MBF quantification (Bratis et al. 2013), and there are various perfusion models and radiotracers available for research and clinical application (Maddahi and Packard 2014). ^13^N-Ammonia was employed as the radiotracer of choice, as it has an intermediate half-life (9.96 min) which facilitates its practical use and has very good image quality given its short positron range and retention in myocardial tissue (Maddahi and Packard 2014). However, ^13^N-Ammonia does not have linear tracer uptake-myocardial blood flow kinetics, particularly at high flow rates, and exhibits 85% myocardial extraction fraction in vivo (Maddahi and Packard 2014). The ideal PET perfusion radiotracer is ^15^O-Water as it is metabolically inert, freely diffusible, with 100% extraction fraction and has linear tracer kinetics with near perfect correlation between tracer uptake and MBF (Maddahi and Packard 2014). Nevertheless, ^13^N-Ammonia MBF quantification has been validated against the gold standard PET perfusion tracer ^15^O-Water (Bol et al. 1993; Nitzsche et al. 1996). In addition, whilst the quantification of absolute MBF with ^13^N-Ammonia has good diagnostic accuracy (Gould et al. 1986), different published models and software packages for the quantification of absolute MBF with ^13^N-Ammonia exist (DeGrado et al. 1996; Hutchins et al. 1990; Nesterov et al. 2009; Adachi et al. 2007; PMOD 2018). De Grado et al. propose a one-tissue compartment model with the use of the first 4 min after injection of tracer to avoid the effects of metabolite build up and washout (DeGrado et al. 1996). Hutchins et al. propose a two-tissue compartment model in which a second compartment accounts for the build-up of metabolically trapped ^13^N-glutamine (metabolite of ^13^N-Ammonia) with rate constants between each compartment (Hutchins et al. 1990). This current study utilized a one tissue compartment model for the perfusion phantom which does not have a second compartment or production of secondary metabolites. As such, we also investigated absolute MBF values in the volunteer study using the one-compartment De Grado model. Using this approach simplified the quantification process and allowed a direct comparison of a one-tissue compartment model comparable between CMR and PET MBF in phantoms and volunteers.

Another important factor that may explain the differences observed of MBF on a per territory basis relates to quantification of MBF of a full 3D volumetric dataset with PET, whereas in CMR, three distinct anatomical 8 mm slices of the heart at the base, mid and apex are acquired.

During routine CMR image acquisition, patients are instructed to breath hold for intermittent periods during the course of the scan. However, during routine PET imaging, patients are instructed to breathe normally. Thus, instructing patients to breath hold during simultaneous PET-MR acquisition may interrupt the rhythmic respiratory pattern, and it is unknown what the effect of this is on the PET data. In order to minimize this as a possible confounder on the PET data, we used a free breathing CMR perfusion sequence in this study, as opposed to isolated breath holds during PET acquisition.

### Limitations

The human circulatory system is a highly complex system composed of the large vessels, pulsatile blood flow, the microcirculation, cardiac myocytes, plasma, and different compartments such as the intra and extracellular space, which are all tightly regulated by autoregulatory systems to maintain homeostatic conditions. In its current form, the perfusion phantom cannot replicate the intricacies of the physiological processes of the human body.

In addition, the exponential decrease of radiotracer in the perfusion phantom may have a different profile for washout phase for the tracer kinetic system when tracer is continuously washed out of the system in the perfusion phantom compared to the human circulatory system. However, a fixed cardiac output and heart rate similar to normal human physiology was employed for the phantom.

Furthermore, the fluid used in the phantom was normal saline, which has different T1 values than blood in the human circulatory system. Nevertheless, the perfusion phantom is free from respiratory artefact, ECG mis-triggering and allows preclinical development in a highly controlled environment that mimics the uptake of tracer into a compartment akin to that formed between the blood in the left ventricular cavity and the myocardial tissue. Future developments of the perfusion phantom into a multicompartmental model may provide incremental steps to simulation of physiological conditions that more closely align with the complexities of the human circulatory system, that may also allow the modelling of multi-compartment tracer kinetics.

Finally, the number of repetitions of the study was limited, and this is in part due to the build-up of radioactivity in the waste water tank, with a finite volume. The sample size is relatively small, although this study serves as proof of principle of simultaneous hybrid PET-MR perfusion prior to further clinical evaluation.

### Future work

Having demonstrated the feasibility of simultaneous hybrid PET-MR perfusion imaging in phantom and volunteers, the next step is to undertake a clinical study in a cohort of patients with suspected CAD.

Several studies have compared the diagnostic accuracy of quantitative CMR for the detection of significant CAD defined by invasive coronary angiography. A clinical validation study of CMR perfusion against ^15^O-Water PET that determines absolute MBF, rather than fractional flow reserve measurements (which determine the haemodynamic significance of epicardial coronary stenosis) is a more appropriate comparator. ^15^O-Water is the gold standard perfusion tracer, and despite its practical challenges due to short half-life, is the ideal perfusion tracer to compare against novel methods for CMR perfusion which allow real-time, at point of care, fully automated in line quantification and shows promise towards implementation into clinical routine (Kellman et al. 2017). Such a study may facilitate clinical and scientific acceptance for CMR perfusion as a feasible alternative for MBF measurements which may allow widespread use without the need for ionizing radiation and expensive radiochemistry facilities.

## Conclusion

Hybrid PET-MR perfusion imaging with ^13^N-Ammonia and gadolinium contrast is feasible. The correlation and agreement of PET and CMR-derived MBF values was high in the perfusion phantom and moderate in healthy volunteers. Undertaking hybrid PET-MR perfusion imaging with a dual bolus set up is complex and challenging, and a single bolus dual sequence approach may be more practical and preferable. Future evaluation in a cohort of patients with CAD is warranted.

## Additional file

Additional file 1: Details of the perfusion phantom. (DOCX 513 kb)

## Abbreviations

AC: Attenuation Correction
AIF: Arterial input function
CAD: Coronary artery disease
CMR: Cardiac magnetic resonance
CT: Computed tomography
MBF: Myocardial blood flow
MR-IMPACT: Magnetic Resonance Imaging for Myocardial Perfusion Assessment in Coronary Artery Disease Trial
OSEM: Ordered subset expectation maximization
PET: Positron emission tomography
ROI: Region of interest

## References

[CR1] Adachi I, Gaemperli O, Valenta I, Schepis T, Siegrist PT, Treyer V (2007). Assessment of myocardial perfusion by dynamic O-15-labeled water PET imaging: validation of a new fast factor analysis. J Nucl Cardiol.

[CR2] Bassingthwaighte JB, Wang CY, Chan IS (1989). Blood-tissue exchange via transport and transformation by capillary endothelial cells. Circ Res.

[CR3] Bol A, Melin JA, Vanoverschelde JL, Baudhuin T, Vogelaers D, De Pauw M (1993). Direct comparison of [13N]ammonia and [15O]water estimates of perfusion with quantification of regional myocardial blood flow by microspheres. Circulation..

[CR4] Bratis K, Mahmoud I, Chiribiri A, Nagel E (2013). Quantitative myocardial perfusion imaging by cardiovascular magnetic resonance and positron emission tomography. J Nucl Cardiol.

[CR5] Brown LAE, Onciul SC, Broadbent DA, Johnson K, Fent GJ, Foley JRJ (2018). Fully automated, inline quantification of myocardial blood flow with cardiovascular magnetic resonance: repeatability of measurements in healthy subjects. J Cardiovasc Magn Reson.

[CR6] Cerqueira MD, Weissman NJ, Dilsizian V, Jacobs AK, Kaul S, Laskey WK (2002). Standardized myocardial segmentation and nomenclature for tomographic imaging of the heart. Circulation..

[CR7] Chang C-Y, Hung G-U, Hsu B, Yang B-H, Chang C-W, Hu L-H et al (2018) Simplified quantification of 13N-ammonia PET myocardial blood flow: a comparative study with the standard compartment model to facilitate clinical use. J Nucl Cardiol. 10.1007/s12350-018-1450-1 10.1007/s12350-018-1450-1 30324328

[CR8] Chiribiri A, Schuster A, Ishida M, Hautvast G, Zarinabad N, Morton G (2013). Perfusion phantom: an efficient and reproducible method to simulate myocardial first-pass perfusion measurements with cardiovascular magnetic resonance. Magn Reson Med.

[CR9] Christian TF, Rettmann DW, Aletras AH, Liao SL, Taylor JL, Balaban RS (2004). Absolute myocardial perfusion in canines measured by using dual-bolus first-pass MR imaging. Radiology.

[CR10] DeGrado TR, Hanson MW, Turkington TG, Delong DM, Brezinski DA, Vallee JP (1996). Estimation of myocardial blood flow for longitudinal studies with 13N-labeled ammonia and positron emission tomography. J Nucl Cardiol.

[CR11] Engblom H, Xue H, Akil S, Carlsson M, Hindorf C, Oddstig J (2017). Fully quantitative cardiovascular magnetic resonance myocardial perfusion ready for clinical use: a comparison between cardiovascular magnetic resonance imaging and positron emission tomography. J Cardiovasc Magn Reson.

[CR12] Farhad H, Dunet V, Bachelard K, Allenbach G, Kaufmann PA, Prior JO (2013). Added prognostic value of myocardial blood flow quantitation in rubidium-82 positron emission tomography imaging. Eur Heart J Cardiovasc Imaging.

[CR13] Fritz-Hansen T, Hove JD, Kofoed KF, Kelbaek H, Larsson HB (2008). Quantification of MRI measured myocardial perfusion reserve in healthy humans: a comparison with positron emission tomography. J Magn Reson Imaging.

[CR14] Gatehouse PD, Elkington AG, Ablitt NA, Yang GZ, Pennell DJ, Firmin DN (2004). Accurate assessment of the arterial input function during high-dose myocardial perfusion cardiovascular magnetic resonance. J Magn Reson Imaging.

[CR15] Gould KL, Goldstein RA, Mullani NA, Kirkeeide RL, Wong WH, Tewson TJ (1986). Noninvasive assessment of coronary stenoses by myocardial perfusion imaging during pharmacologic coronary vasodilation. VIII. Clinical feasibility of positron cardiac imaging without a cyclotron using generator-produced rubidium-82. J Am Coll Cardiol.

[CR16] Herzog BA, Husmann L, Valenta I, Gaemperli O, Siegrist PT, Tay FM (2009). Long-term prognostic value of 13N-ammonia myocardial perfusion positron emission tomography added value of coronary flow reserve. J Am Coll Cardiol.

[CR17] Hsu LY, Jacobs M, Benovoy M, Ta AD, Conn HM, Winkler S (2018). Diagnostic performance of fully automated pixel-wise quantitative myocardial perfusion imaging by cardiovascular magnetic resonance. JACC Cardiovasc Imaging.

[CR18] Hussain ST, Paul M, Plein S, McCann GP, Shah AM, Marber MS (2012). Design and rationale of the MR-INFORM study: stress perfusion cardiovascular magnetic resonance imaging to guide the management of patients with stable coronary artery disease. J Cardiovasc Magn Reson.

[CR19] Hutchins GD, Schwaiger M, Rosenspire KC, Krivokapich J, Schelbert H, Kuhl DE (1990). Noninvasive quantification of regional blood flow in the human heart using N-13 ammonia and dynamic positron emission tomographic imaging. J Am Coll Cardiol.

[CR20] Ishida M, Schuster A, Morton G, Chiribiri A, Hussain S, Paul M (2011). Development of a universal dual-bolus injection scheme for the quantitative assessment of myocardial perfusion cardiovascular magnetic resonance. J Cardiovasc Magn Reson.

[CR21] Jerosch-Herold M (2010). Quantification of myocardial perfusion by cardiovascular magnetic resonance. J Cardiovasc Magn Reson.

[CR22] Jerosch-Herold M, Wilke N, Stillman AE (1998). Magnetic resonance quantification of the myocardial perfusion reserve with a Fermi function model for constrained deconvolution. Med Phys.

[CR23] Kellman P, Hansen MS, Nielles-Vallespin S, Nickander J, Themudo R, Ugander M (2017). Myocardial perfusion cardiovascular magnetic resonance: optimized dual sequence and reconstruction for quantification. J Cardiovasc Magn Reson.

[CR24] Kitkungvan D, Johnson NP, Roby AE, Patel MB, Kirkeeide R, Gould KL (2017). Routine clinical quantitative rest stress myocardial perfusion for managing coronary artery disease: clinical relevance of test-retest variability. JACC Cardiovasc Imaging.

[CR25] Knott KD, Camaioni C, Ramasamy A, Augusto JA, Bhuva AN, Xue H et al (2019) Quantitative myocardial perfusion in coronary artery disease: a perfusion mapping study. J Magn Reson Imaging. 10.1002/jmri.26668. Epub ahead of print 10.1002/jmri.26668 PMC6767569 30684288

[CR26] Kotecha T, Martinez-Naharro A, Boldrini M, Knight D, Hawkins P, Kalra S et al (2019) Automated pixel-wise quantitative myocardial perfusion mapping by CMR to detect obstructive coronary artery disease and coronary microvascular dysfunction: validation against invasive coronary physiology. JACC Cardiovasc Imaging. Epub ahead of print 10.1016/j.jcmg.2018.12.022 PMC8414332 30772231

[CR27] Kunze Karl P., Nekolla Stephan G., Rischpler Christoph, Zhang Shelley HuaLei, Hayes Carmel, Langwieser Nicolas, Ibrahim Tareq, Laugwitz Karl-Ludwig, Schwaiger Markus (2018). Myocardial perfusion quantification using simultaneously acquired 13 NH3 -ammonia PET and dynamic contrast-enhanced MRI in patients at rest and stress. Magnetic Resonance in Medicine.

[CR28] Larghat AM, Maredia N, Biglands J, Greenwood JP, Ball SG, Jerosch-Herold M (2013). Reproducibility of first-pass cardiovascular magnetic resonance myocardial perfusion. J Magn Reson Imaging.

[CR29] Lassen ML, Rasul S, Beitzke D, Stelzmuller ME, Cal-Gonzalez J, Hacker M et al (2017) Assessment of attenuation correction for myocardial PET imaging using combined PET/MRI. J Nucl Cardiol. Epub ahead of print 10.1007/s12350-017-1118-2 PMC6660490 29168158

[CR30] Lee DC, Johnson NP (2009). Quantification of absolute myocardial blood flow by magnetic resonance perfusion imaging. JACC Cardiovasc Imaging.

[CR31] Maddahi J, Packard RR (2014). Cardiac PET perfusion tracers: current status and future directions. Semin Nucl Med.

[CR32] Martinez-Moller A, Souvatzoglou M, Delso G, Bundschuh RA, Chefd'hotel C, Ziegler SI (2009). Tissue classification as a potential approach for attenuation correction in whole-body PET/MRI: evaluation with PET/CT data. J Nucl Med.

[CR33] Miller CA, Naish JH, Ainslie MP, Tonge C, Tout D, Arumugam P (2014). Voxel-wise quantification of myocardial blood flow with cardiovascular magnetic resonance: effect of variations in methodology and validation with positron emission tomography. J Cardiovasc Magn Reson.

[CR34] Morton G, Chiribiri A, Ishida M, Hussain ST, Schuster A, Indermuehle A (2012). Quantification of absolute myocardial perfusion in patients with coronary artery disease: comparison between cardiovascular magnetic resonance and positron emission tomography. J Am Coll Cardiol.

[CR35] Morton G, Ishida M, Schuster A, Hussain S, Schaeffter T, Chiribiri A (2012). Perfusion cardiovascular magnetic resonance: comparison of an advanced, high-resolution and a standard sequence. J Cardiovasc Magn Reson.

[CR36] Nazir MS, Ismail TF, Reyes E, Chiribiri A, Kaufmann PA, Plein S (2018). Hybrid positron emission tomography-magnetic resonance of the heart: current state of the art and future applications. Eur Heart J Cardiovasc Imaging.

[CR37] Nesterov SV, Han C, Maki M, Kajander S, Naum AG, Helenius H (2009). Myocardial perfusion quantitation with 15O-labelled water PET: high reproducibility of the new cardiac analysis software (Carimas). Eur J Nucl Med Mol Imaging.

[CR38] Nitzsche EU, Choi Y, Czernin J, Hoh CK, Huang SC, Schelbert HR (1996). Noninvasive quantification of myocardial blood flow in humans. A direct comparison of the [13N]ammonia and the [15O]water techniques. Circulation..

[CR39] O’Doherty J, Chalampalakis Z, Schleyer P, Nazir MS, Chiribiri A, Marsden PK (2017). The effect of high count rates on cardiac perfusion quantification in a simultaneous PET-MR system using a cardiac perfusion phantom. EJNMMI Phys.

[CR40] Otton J, Morton G, Schuster A, Bigalke B, Marano R, Olivotti L (2013). A direct comparison of the sensitivity of CT and MR cardiac perfusion using a myocardial perfusion phantom. J Cardiovasc Comput Tomogr.

[CR41] Parkka JP, Niemi P, Saraste A, Koskenvuo JW, Komu M, Oikonen V (2006). Comparison of MRI and positron emission tomography for measuring myocardial perfusion reserve in healthy humans. Magn Reson Med.

[CR42] Plein S, Greenwood J, Ridgway JP (2015). Cardiovascular MR Manual.

[CR43] PMOD. http://www.pmod.com/files/download/v31/doc/pkin/2283.htm. Accessed 29th September 2018 [Available from: http://www.pmod.com/files/download/v31/doc/pkin/2283.htm]

[CR44] Zarinabad N, Chiribiri A, Hautvast GL, Ishida M, Schuster A, Cvetkovic Z (2012). Voxel-wise quantification of myocardial perfusion by cardiac magnetic resonance. Feasibility and methods comparison. Magn Reson Med.

[CR45] Zierler KL (1962). Theoretical basis of indicator-dilution methods for measuring flow and volume. Circ Res.

