# Supplementary material for: Simultaneous 13N-Ammonia and gadolinium first-pass myocardial perfusion with quantitative hybrid PET-MR imaging: a phantom and clinical feasibility study
Source: Eur J Hybrid Imaging. 2019 Sep 3;3:15. doi: 10.1186/s41824-019-0062-6 (PMC6718374; doi:10.1186/s41824-019-0062-6)

**Additional file 1**

A schematic diagram of the perfusion phantom is provided in Figure 6. The system is composed of four main parts:

- a water pump that generates the flow of fluid across the phantom circuitry
- a compact unit that mimics the thoracic great vessels (inferior vena cava, pulmonary artery, pulmonary vein, thoracic aorta) the four cardiac chambers (left and right atria and ventricles) and the myocardium of a 60kg human. The unit can be inserted into the bore of most commercially available clinical MRI, PET and Computer Tomography (CT) scanners and is MRI compatible.
- a computer control unit which operates a series of highly controlled electronic flowmeters that control flow to the phantom. The flowmeters can be remotely at distance from the circuitry, controlled by a handheld computer tablet operated by wireless connection.
- a large water tank that acts as a storage for waste fluid for radioactive tracers.


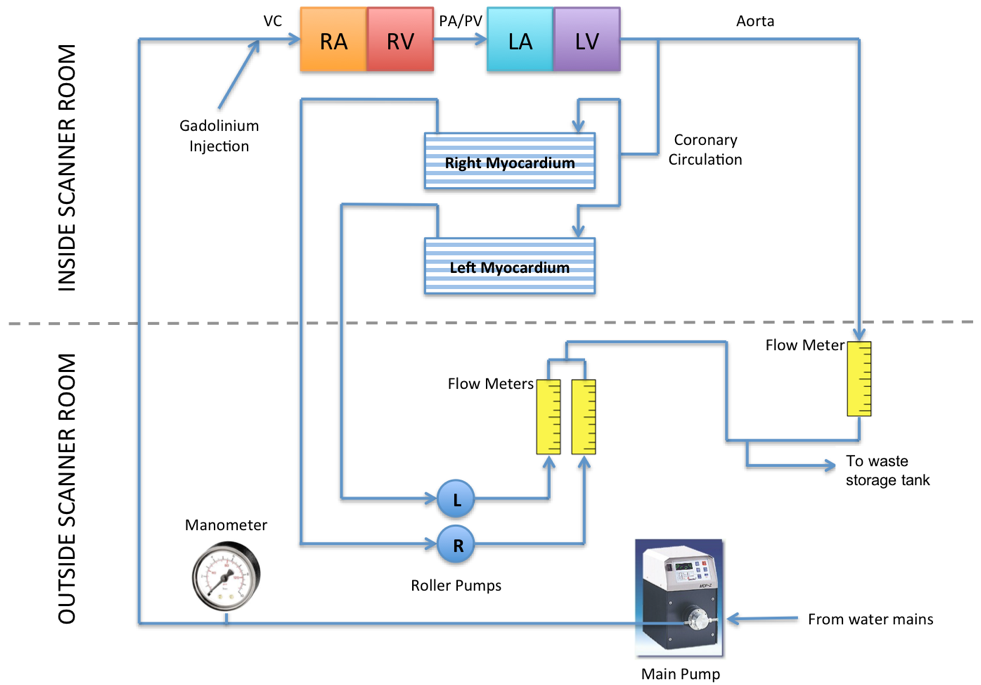


Figure 6. Schematic diagram of the perfusion phantom.

VC: vena cava, RA: right atrium, RV: right ventricle, PA: pulmonary artery, PV: pulmonary vein. Reproduced from (18).

The system is intricately controlled from roller pump flow meters that ‘feed-forward’ the flow rates, and the return flow meters ‘feed-back’ the measured flow rates to ensure continuous internal validation of the system.

A 3D volume rendered image reconstructed following high resolution CMR imaging of the myocardial filter with sample of the image plane (blue) is presented below.


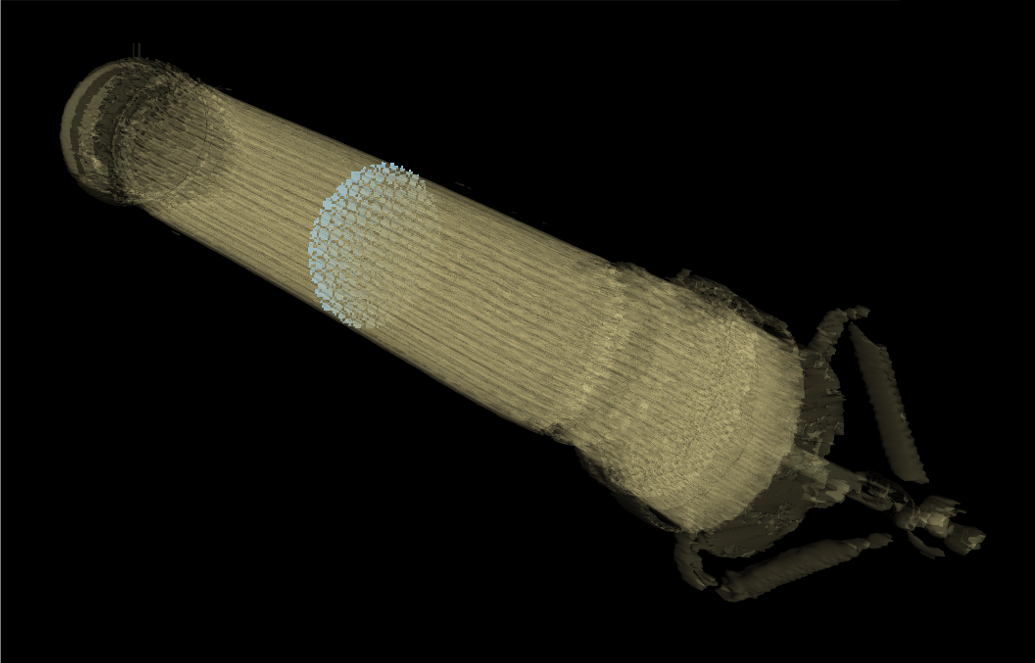

Supplement: Supplementary file 1 — Details of the perfusion phantom. (DOCX 513 kb) [file 41824_2019_62_MOESM1_ESM.docx]
